# Seasonal and spatial variability of the partial pressure of carbon dioxide in the human-impacted Seine River in France

**DOI:** 10.1038/s41598-018-32332-2

**Published:** 2018-09-18

**Authors:** Audrey Marescaux, Vincent Thieu, Alberto Vieira Borges, Josette Garnier

**Affiliations:** 1 Sorbonne Université, Centre National de la Recherche Scientifique, Institut Pierre Simon Laplace, UMR, 7619 METIS Paris, France; 2 Université de Liège, Unité d’Océanographie Chimique, Liège, Belgium

## Abstract

Carbon evasion from rivers is an important component of the global carbon cycle. The intensification of anthropogenic pressures on hydrosystems requires studies of human-impacted rivers to identify and quantify the main drivers of carbon evasion. In 2016 and 2017, four field campaigns were conducted in the Seine River network characterized by an intensively cropped and highly populated basin. We measured partial pressures of carbon dioxide (pCO_2_) in streams or rivers draining land under different uses at different seasons. We also computed pCO_2_ from an existing data set (pH, water temperature and total alkalinity) going back until 1970. Here we report factors controlling pCO_2_ that operate at different time and space scales. In our study, the Seine River was shown to be supersaturated in CO_2_ with respect to the atmospheric equilibrium, as well as a source of CO_2_. Our results suggest an increase in pCO_2_ from winter to summer in small streams draining forests (from 1670 to 2480 ppm), croplands (from 1010 to 1550 ppm), and at the outlet of the basin (from 2490 to 3630 ppm). The main driver of pCO_2_ was shown to be dissolved organic carbon (DOC) concentrations (R^2^ = 0.56, n = 119, p < 0.05) that are modulated by hydro-climatic conditions and groundwater discharges. DOC sources were linked to land use and soil, mainly leaching into small upstream streams, but also to organic pollution, mainly found downstream in larger rivers. Our long-term analysis of the main stream suggests that pCO_2_ closely mirrors the pattern of urban water pollution over time. These results suggest that factors controlling pCO_2_ operate differently upstream and downstream depending on the physical characteristics of the river basin and on the intensity and location of the main anthropogenic pressures. The influence of these controlling factors may also differ over time, according to the seasons, and mirror long term changes in these anthropogenic pressures.

## Introduction

Globally, streams and rivers are estimated to contribute significantly to carbon budgets, with two recent studies estimating carbon dioxide (CO_2_) emissions in the order of $\,{0.65}_{-0.17}^{+0.20}$ PgC yr^−1^ and 1.80 ± 0.25 PgC yr^−1^ ^1,2^. This wide range underlines continuing uncertainty, and regional studies are thus needed to provide a better description of the processes driving these carbon fluxes.

Excessive or deficient CO_2_ concentration in water with respect to atmospheric equilibrium determines whether inland waters are a CO_2_ source or sink. In the majority of river drainage networks, the ratio of primary production to respiration is less than 1, contributing to carbon evasion from inland waters to the atmosphere^3–5^. CO_2_ supersaturation in waters with respect to the CO_2_ atmospheric equilibrium can result from the bacterial mineralization of biodegradable organic material exported from soils and autochthonous production as well as inorganic carbon imports from soils (weathering of the bedrock, acidification of buffered waters, etc.)^6^. In rivers with extensive wetlands (flooded forests and floating macrophytes), lateral DOC enhancing mineralization in the river channel’s and CO_2_ transports are particularly important^7–10^. In addition, CO_2_ in rivers can be transferred from groundwaters^11^.

Under the temperate European climate, partial pressure of CO_2_ (pCO_2_) values in rivers display significant variability related to land use, lithology and hydrological conditions. For example, in France, pCO_2_ levels of around 284 ppm were measured in the Loire (croplands)^12^, from 1604 to 6546 ppm in the podzolized Arcachon catchment’s streams, with higher values when discharge is low^13^, and 2292 ppm in the carbonate-rock-dominated Meuse watershed, which is mostly covered by forests, grasslands and croplands^12^. A recent study of the Meuse River^14^ revealed marked variations in pCO_2_ (34 to 10,033 ppm) the higher values being associated with watersheds dominated by agriculture and lower values with forested watersheds. CO_2_ undersaturation with respect to the atmospheric equilibrium has been demonstrated in the upstream part of the Danube River basin related to photosynthetic uptake in summer^15^.

In the Seine River basin, previous carbon investigations focused on organic carbon^16^, methane emissions from soils, livestock and the river network^17^ or on benthic respiration^18^ and ecological status based on the production/respiration ratio^4^. These studies did not specifically address CO_2_ concentrations. Our objective here was to quantify pCO_2_ in the Seine River, using both recent *in situ* measurements and calculations based on long time series of existing data, in order to evaluate the distribution of pCO_2_ and CO_2_ evasion in the drainage network, and to identify the major factors controlling pCO_2_.

## Materials and Methods

### Study site

**Figure 1:**
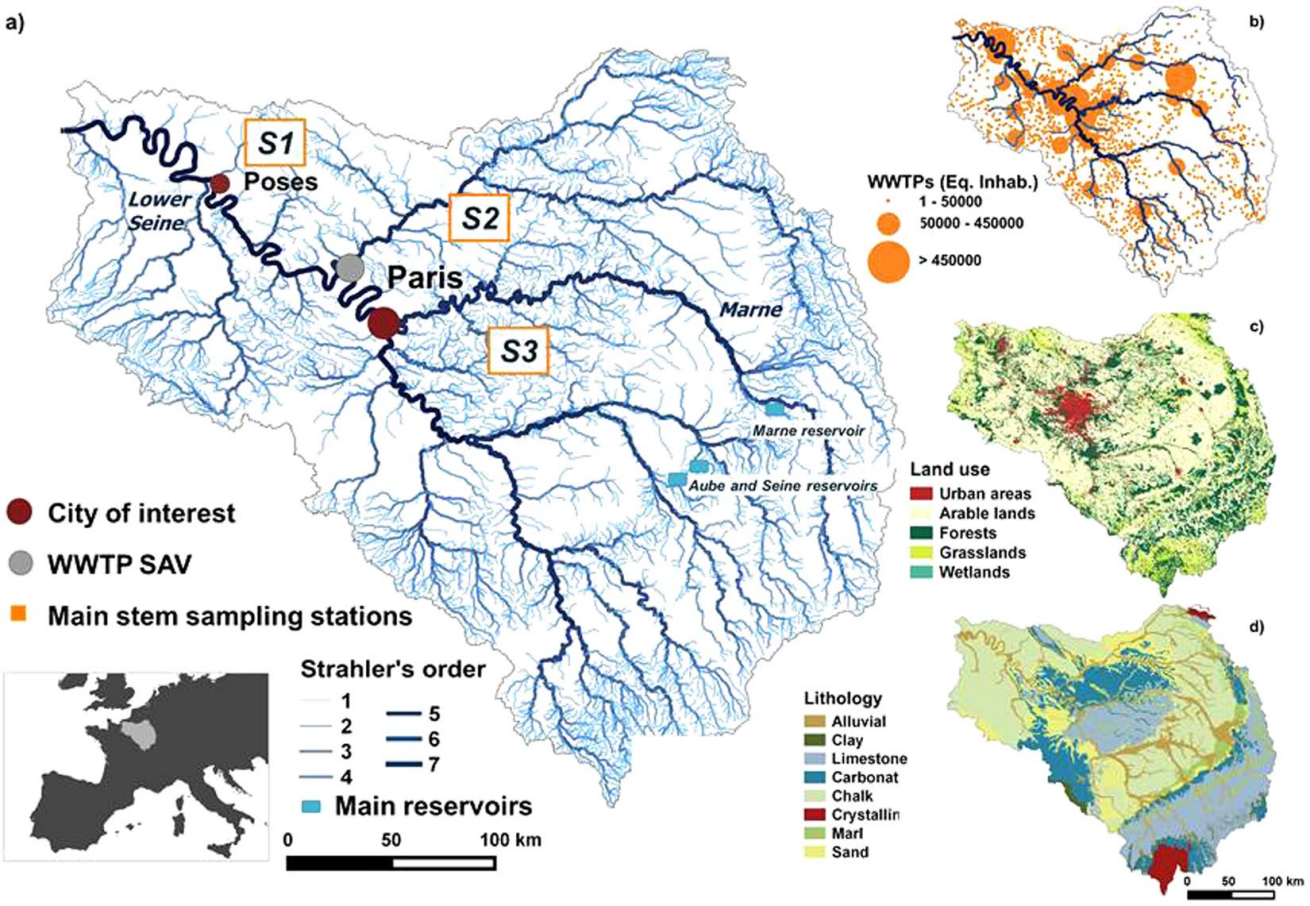
Maps of the Seine river basin created using QGIS software^60^. (**a**) The hydrographic network with Strahler orders from 1 to 7, main urban centers (carmine red dots), small stream sampling zones (S1, S2 and S3), main stream sampling sites (orange squares along the lower Seine and the Marne rivers, the three main reservoirs (blue squares) and the main wastewater treatment plant (grey dot). (**b**) Wastewater treatment plants in the Seine basin mapped according to their treatment capacity (AESN 2012); (**c**) Land uses in the Seine basin (CLC database, IFEN 2012)^61^; (**d**) Lithology of the Seine basin (Albinet, 1967)^62^.

The Seine River watershed, located in northern France, covers an area of 76,750 km^2^ with a median slope of 2.2° and 89.5% of its area is less than 300 m A.S.L. It has a pluvio-oceanic regime and its annual water flow in the period 2013–2016 averaged 550 m^3^ s^−1^ at the river outlet at Poses (Fig. 1). The Poses monitoring station, located at a navigation dam, is the most downstream station not subject to the dynamic influence of the tidal estuary. Low water flows (<300 m^3^ s^−1^) are generally observed from March to November, while high flows (>800 m^3^ s^−1^) occur in winter, from December to February (data provided by the HYDRO database, http://www.hydro.eaufrance.fr, 2018).

Three major diverted reservoirs (Fig. 1a) located in the upstream part of the basin (the Marne reservoir, the Aube reservoir and the Seine reservoir) were built to reduce high water events in winter, and to sustain the flow in late summer. They have a combined storage capacity of 800 10^6^ m^3^ and a surface area of 65 km² ^19^.

The basin is densely populated (~230 inhabitants km^−2^) mostly concentrated in the Paris conurbation (12.4 million inhabitants in 2015) in the central part of the basin. The largest wastewater treatment plant in Europe: Seine Aval, (French acronym SAV) WWTP with a dry weather capacity of 1,500,000 m^3^ d^−1^) is located 50 km downstream of the center of Paris (Fig. 1b). The corresponding effluents account for more than 30% of the organic carbon load of all the WWTPs in the basin^20^. Major upgrading of wastewater treatments of the SAV followed the Urban Wastewater Treatment Directive (1991, 91/271/CEE) among which the addition of flocculation (2000–2003), nitrification (2007) and denitrification (30% in 2007 and 70% in 2012)^21^. The Seine counts about 1700 smaller capacity WWTPs spread throughout the basin (Fig. 1b). The basin comprises 56.8% arable land (mainly under intensive agriculture), 25.8% forests, 9.7% grasslands and 7.0% urban areas (CLC database, IFEN 2012, Fig. 1c). Wetlands have been estimated at between 10.9% and 15.6% of the surface area of the basin^22^. The Strahler stream order^23^ of the main stream of the basin is 6^th^ order for the Marne River and 7^th^ order for the Seine River downstream of Paris (Fig. 1a). The sedimentary basin of the Seine River is characterized by geological formations with low slope gradients resulting in concentric lithology dominated by carbonate and limestone in the central part of the basin, a wide band of Cretaceous chalk and a narrow band of clay followed by Jurassic limestone at the periphery (Fig. 1d).

### Sampling strategy, physical-chemical analysis and direct measurements of pCO2

We sampled 30 sites in streams chosen because they mainly drain grasslands, forests and wetlands, croplands, and along the main streams of the Marne River (including in its reservoir) and of the lower Seine (Fig. 1a, exact locations in Supplementary Material 1). Sampling campaigns were carried out in four contrasting hydro-climatological periods. Water discharges were measured at the outlet of the basin (Poses) and temperatures were measured at each sampling site in winter from February 22 to March 10, 2016, (1030 m^3^ s^−1^, 6.9 °C on average), in summer/autumn from September 7 to 14, 2016, (270 m^3^ s^−1^, 18.8 °C), spring from March 14 to 23, 2017, (580 m^3^ s^−1^, 9.9 °C) as well as during a spring flood event that was exceptional in its timing, from May 23 to June 2, 2016, (1500 m^3^ s^−1^, 13.0 °C, at sampling time with a maximum discharge reaching 2000 m^3^ s^−1^ at the river outlet, at Poses). The field campaigns were assumed to be key seasonal and hydrological periods and were conducted in areas representing the main types of land use in the Seine River basin.

Direct pCO_2_ measurements were based on the syringe headspace technique^12,24^ combined with non-dispersive infrared gas analysis (IRGA) (Li-cor® models 820 or 840; accuracy <3% of reading). Calibration was performed using CO_2_ concentration of 799 ppm and CO_2_-free dinitrogen. Four syringes coupled with three-way valves were filled directly in the stream or river, each replicate containing 30 mL of river water and 30 mL of atmospheric air. Closed syringes were continuously shaken for 10 min to equilibrate CO_2_ concentrations of gas and water. The equilibrated gas was injected into the IRGA and water temperature inside the syringe was measured. The first injection served as a purge and the other three were used for pCO_2_ measurements. The initial pCO_2_ in water was computed based on the pCO_2_ measured in the equilibrated air of the syringe and in the atmospheric air, and Henry’s law accounting for the water temperature in the syringe and *in situ*.

Simultaneously, 2 L water chemistry high-density polyethylene sampling bottles were used to collect samples from bridges over the main stream, and along the banks of smaller streams. Water temperature, pH, dissolved oxygen and conductivity were measured in the field using a multi-parameter probe (YSI® 6600 V2, accuracy ± 0.2 units). Calibrations of the probe were completed with pH 7 and pH 4 buffers for pH (NBS Scale), potassium chloride (KCl) electrolyte solution for dissolved oxygen and 10 mS cm^−1^ standard for conductivity. In the laboratory, water subsamples were filtered on combusted filters for 4 h at 500 °C: GF/F 0.7 µm, 25 mm) to analyze particulate inorganic and organic carbon (PIC and POC, respectively). Filtrates enabled measurement of dissolved inorganic and organic carbon (DIC and DOC) concentrations and total alkalinity (TA). One milliliter of sulfuric acid (3 M) was added to the DOC samples to stop biological reactions. Dissolved inorganic and organic carbon were analyzed with a TOC analyzer (Aurora 1030). Nongaseous DIC analyses required acidification of the filtrated sample by adding sodium persulfate reagents (100 g L^−1^) to dissociate the carbonates in the CO_2_ that were detected by an IRGA. The inorganic free sample was used for DOC measurements. DOC was measured by wet oxidation by adding 10% phosphoric acid oxide followed by high temperature (680 °C) catalytic combustion, and then detected using an NDIR technique. TA (µmol kg^−1^) was analyzed using an automatic titrator (TitroLine® 5000) on three 20 mL replicates of filtered water (GF/F: 0.7 μm), with hydrochloric acid (0.1 M).

Values of total suspended solids (TSS) were determined as the weight of material retained on a Whatman GF/F membrane per volume unit after drying the filter for 2 h at 120 °C. Chlorophyll *a* concentrations (Chl. a) were determined according to Lorenzen^25^.

Aquifer waters were also sampled during the same periods. Groundwater was pumped from the piezometers using a peristaltic pump. Before the samples were collected, the piezometers were emptied by flushing to remove the standing water (5–10 L in each piezometer)^26^. The same variables were measured or analyzed, except Chl. a.

### pCO_2_ calculations from existing data

pCO_2_ were computed with the CO_2_SYS software^27^ using the water temperature and two of the three following measurements: pH, TA and DIC. In contrast to DIC, TA is often measured by the French water authorities *Agence de l’Eau Seine Normandie* (French acronym AESN, http://www.eau-seine-normandie.fr/, 2018), and thus were preferred to compute pCO_2_ in combination with pH and water temperatures. The carbonate dissociation constants (K1 and K2) applied were from Millero^28^ with zero salinity.

**Figure 2:**
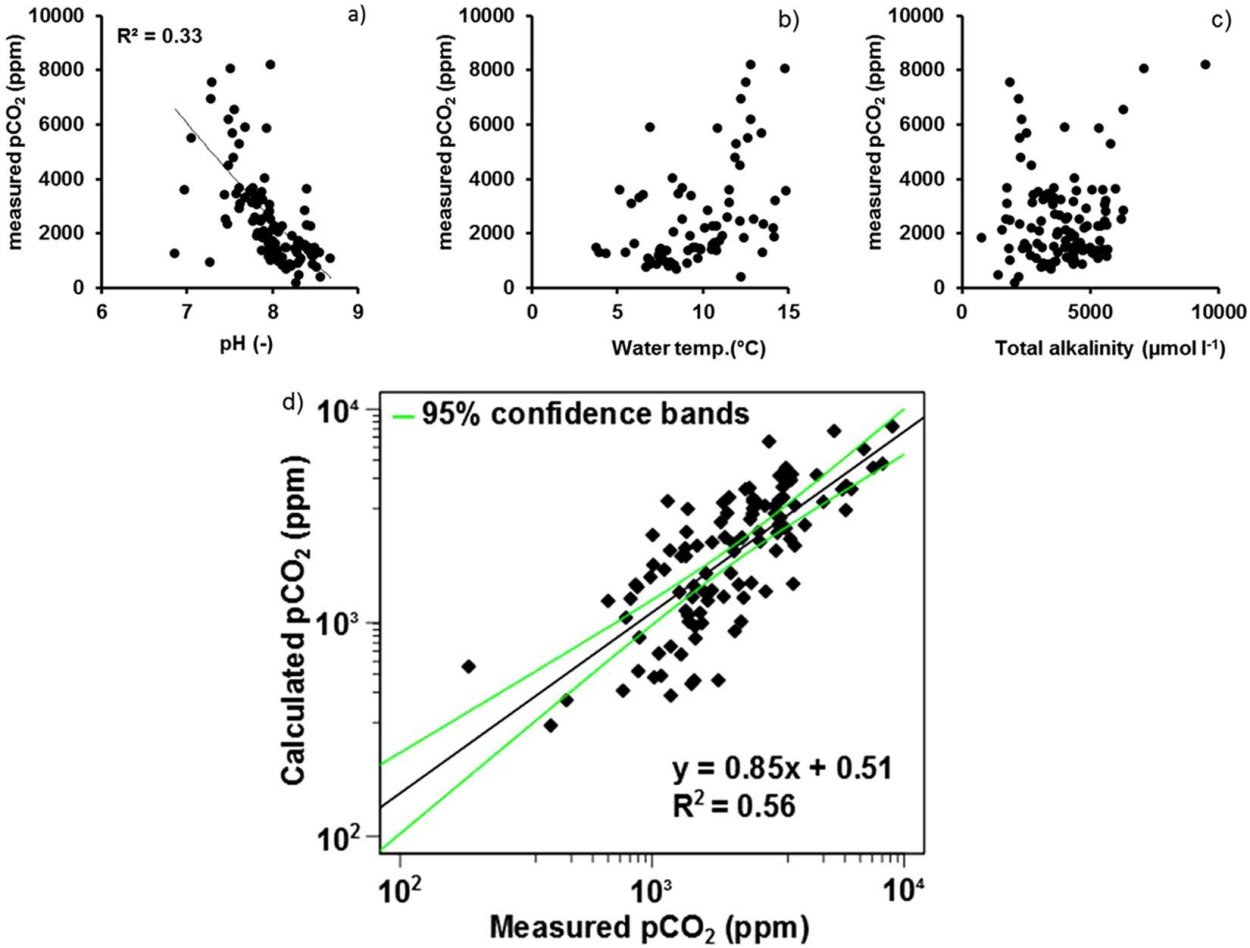
Measured pCO_2_ vs. (**a**) pH (NBS scale); (**b**) water temperature (Water temp.); (**c**) total alkalinity; (**d**) comparison of calculated pCO_2_ (using pH, temperature, alkalinity) and direct measurements of pCO_2_. Green lines represent the 95% confidence intervals.

During our field campaigns (winter 2016, spring 2017, spring flood 2016, summer/autumn 2016, see *previous section*), we systematically combined direct measurements of pCO_2_ with measurements of water temperature, pH and TA (130 samples). We found a positive relationship between the pCO_2_ values directly measured during our field campaigns and those calculated using water temperatures, pH and TA (Fig. 2). This relationship was then used to correct possible bias^12^ of pCO_2_ values calculated with CO2SYS program.

We also used the database (42,108 data with simultaneous water temperatures, pH and TA measured between 1971 and 2015) provided by the French water authorities (AESN) to compute and analyze the pCO_2_ dynamics in the Seine basin in space and over time since the 1970s. These pCO_2_ data series were corrected by the relationship previously established and then averaged by months and years at each monitoring station. Within this timeframe (1970–2015), two periods of interest (ii) 1989–1991 (92 monitoring stations) and (ii) 2013–2015 (234 monitoring stations) were defined as representative of the changes that occurred recently in the Seine river basin. The former period (1989–1991) represents the period of highest organic pollution from WWTPs, only treated by activated sludge. The most recent period (2013–2015) illustrates the state after a full implementation of the Urban Wastewater Treatment Directive (1991, 91/271/CEE), including the reduction of point sources of organic carbon (industrial and domestic) discharge into the river, as well as phosphorus and nitrogen^20,21,29^. In addition, we assessed the spatial variability of pCO_2_ along the main stream of the Seine River, comparing the concentrations of the most important effluents up- and downstream (Paris and Poses stations, Fig. 1) of the SAV WWTP. We also computed pCO_2_ at a constant temperature of 10 °C (pCO2@10 °C) downstream of the SAV WWTP to show the impact of solubility on pCO_2_ (see results section: “*Long-term pCO*_2_ *variability (1970–*2*015*)*”*).

### Determination of gas transfer velocities

Raymond *et al*.^30^ pointed out that gas transfer velocity equations including slope and water velocity enable easy measurements and recommended the use of these equations at large spatial scales. We selected the equation requiring only the slope and the water velocity and that had the highest squared-R in the Raymond *et al*.^30^ study (Equation 5 in Table 2 in Raymond *et al*. (2012)) as we wanted to compare the variability of CO_2_ evasion in the basin in space and over time. We used kinematic water viscosity coefficients and Schmidt numbers calculated according to Wanninkhof *et al*.^31^.

The slopes of the streams and rivers were provided by the French water authorities (AESN). Water velocities were estimated from discharge records available at the scale of the whole drainage network for the period 2012–2014. Water temperatures were averaged by season of interest based on our field campaigns (winter, spring and summer/autumn). The *k*-values were calculated by stream order and then aggregated by small streams (Strahler orders (SOs) 1–4) and along the main stream (SOs 5–7). Calculated *k*-values for the spring flood event were based on averaged spring water temperatures associated with measurements of high water flow collected during the exceptional spring flood (May 2016).

According to Wanninkhof^32^, Wilke and Chang^33^ and Raymond *et al*.^30^, the gas transfer velocity ${k}_{C{O}_{2}}$ (m d^−1^) under negligible wind conditions in rivers can be calculated as:

1 $${k}_{C{O}_{2}}={k}_{600}.\sqrt{\frac{600}{S{c}_{C{O}_{2}}(T)}}$$

2 $${k}_{600}=vS\,2841\pm 107+2.02\pm 0.209$$

where *k*_600_ is the gas transfer velocity for a Schmidt number of 600 (m d^−1^),*v* is the water velocity (ms^−1^), *S* the slope (−),107 et 0.209 are the standard deviations of the parameters. *Sc*_*co*2_ (*T*) is the Schmidt number (dimensionless) with the water temperature *T* in Celsius (°*C*) calculated as:

3 $$S{c}_{C{O}_{2}}(T)=1911.1-118.11T+3.4527{T}^{2}-0.04132{T}^{3}$$

The flux (*fCO*_2_, mgC-CO_2_ m^−2^ d^−1^) at the interface of the river and the atmosphere can be calculated as:

4 $$fC{O}_{2}={k}_{C{O}_{2}}([C{O}_{2}]-{[C{O}_{2}]}_{eq})$$

where $\,[C{O}_{2}]$ is the CO_2_ concentration in the water (mgC-CO_2_ m^−3^), and ${[C{O}_{2}]}_{eq}$ is the CO_2_ concentration in equilibrium with atmospheric concentrations (mgC-CO_2_ m^−3^). Annual atmospheric pCO_2_ values measured at Mauna Loa Observatory (Hawaii, U.S.A.) were provided by the NOAA/ESRL (http://www.esrl.noaa.gov/gmd/ccgg/trends/, 2018), Scripps Institution of Oceanography (scrippsco2.ucsd.edu/, 2018). ${k}_{C{O}_{2}}$ (m d^−1^) is the gas transfer velocity (see equation 1).

### Statistical tests

All statistical tests were performed using R software^34^.

Wilcoxon signed-rank tests were used to compare measured pCO_2_ in the four periods, and Kruskal-Wallis tests were used to compare measured pCO_2_ averages for different land uses during each period. A Shapiro-Wilk test was applied to test the normal distribution before performing the linear regression between measured pCO_2_ and calculated pCO_2_. Linear regressions were then performed between pCO_2_ and water quality variables.

## Results

### Measured versus calculated pCO_2_

The streams and rivers sampled during our field campaigns were neutral or basic and carbonate-buffered (Fig. 2a,c), excluding the overestimation of calculated pCO_2_ already shown to be linked to the low buffering capacity of the carbonate system^12^. A logarithmic transformation was performed on both measured and calculated pCO_2_ to obtain normal distribution (Shapiro-Wilk test, *p* > 0.01) to calculate a linear regression. A positive relationship was established (R^2^ = 0.56, n = 130, p < 0.01).

$${\boldsymbol{measured}}\,{{\boldsymbol{p}}}_{{\boldsymbol{C}}{{\boldsymbol{O}}}_{2}}={\bf{1}}{{\bf{0}}}^{(\frac{{\bf{l}}{\bf{o}}{\bf{g}}({\bf{c}}{\bf{a}}{\bf{l}}{\bf{c}}{\bf{u}}{\bf{l}}{\bf{a}}{\bf{t}}{\bf{e}}{\bf{d}}{{\boldsymbol{p}}}_{{\boldsymbol{C}}{{\boldsymbol{O}}}_{2}})-{\bf{0}}{\boldsymbol{.}}51}{{\bf{0}}{\boldsymbol{.}}85})}\,(p < 0.01\,and\,degrees\,of\,freedom=106)$$

### Field campaign dataset overview

Average water temperatures ranged between 6.9 °C and 18 °C which corresponds to the expected seasonal range for the Seine basin (Table 1). pH values were generally neutral to basic, with median values of pH and TA in all streams and rivers ranging respectively from 7.75 to 8.25 and from 3150 µmole l^−1^ to 4350 µmole l^−1^ (see Table 1). Only two acidic pH values measured during the winter in streams draining forests (data not shown). The high total alkalinity measured in all the streams and rivers (Table 1) indicated that waters were carbonate-buffered due to the lithology of the basin, which is dominated by carbonate rocks (Fig. 1d)^35^. Indeed, dissolved inorganic carbon (DIC) concentrations were high (min.: 19.3 mgC l^−1^, Table 1) as was conductivity (median of all campaigns: 0.554 mS cm^−2^), suggesting that bicarbonate ions contributed most to total alkalinity. Dissolved inorganic carbon (DIC) concentrations averaged 52.55 mgC l^−1^ (median: 54.04 mgC l^−1^). Dissolved organic (DOC) concentrations were one order of magnitude lower than those of DIC, the highest being observed in streams draining wetlands (median: 17.25 mgC l^−1^) while streams draining croplands had the lowest concentrations (median: 2.62 mgC l^−1^). Total suspended solids (TSS) were highest (median 20.77 mg l^−1^) in grasslands during the spring flood 2016 (with a median chlorophyll *a* concentration of 27.1 µg l^−1^). Wetlands were mostly undersaturated in oxygen (median: 5.7 mgO_2_ l^−1^ and min. water temperature: 6.1 °C) while the rest of the data set showed oxygenated waters (median: 9.2 mgO_2_ l^−1^; min. – max.: 6.0–15.7 mgO_2_ l^−1^).

**Table 1:** Summary of the field data set. Median, 10^th^ and 90^th^ percentiles pH (measured on the NBS scale), water temperature, total alkalinity, dissolved organic carbon (DOC), dissolved inorganic carbon (DIC), total suspended solids (TSS), chlorophyll a (Chl. a), dissolved oxygen (O_2_) and conductivity. Mean water discharges are showed for seasons at the outlet of the basin.

		pH (−)	Water temp. (°C)	Total alkalinity (µmole l^−1^)	DOC (mgC l^−1^)	DIC (mgC l^−1^)	TSS (mg l^−1^)	Chl. a (µg l^−1^)	O_2_ (mgO_2_ l^−1^)	Conductivity (mS cm^−2^)	Mean water discharges at the outlet (m^3^ s^−1^)
*Winter* 2016	*median*	8.05	6.9	4350	6.9	52.7	22.7	6.6	9.85	0.557	1030
	10^th^*–90*^th^	7.30–8.50	5.0–8.3	2770–5140	2.7–13.8	38.1–64.3	10.3–42.7	2.4–9.6	5.60–13.37	0.327–0.781	
*Spring* 2017	*Median*	8.25	9.9	4255	4	58.6	17	6.1	9.63	0.59	580
	10^th^*–90*^th^	7.82–8.47	8.3–11.0	2375–5475	2.4–14.7	39.6–72.0	8.2–32.8	1.6–25.4	8.88–10.94	0.392–0.678	
*Summer/automn* 2016	*Median*	7.96	18	4037.5	3.5	53.9	7.9	2.3	8.23	0.597	270
	10^th^*–90*^th^	7.83–8.33	15.5–22.6	1872.5–5685	2.0–6.6	28.2–73.6	4.1–45.3	1.1–10.9	9.00–8.23	0.373–0.695	
*Spring flood* 2016	*Median*	7.75	13	3150	11.4	45.7	20.77	12	8.81	0.49	1500
	10^th^*–90*^th^	7.29–7.98	11.9–15.5	1875–5800	3.4–20.9	26.3–66.9	3.2–214.0	2.3–30	5.81–9.58	0.311–0.648	

### Variability in pCO_2_

#### Spatial and seasonal variability of pCO_2_

**Figure 3:**
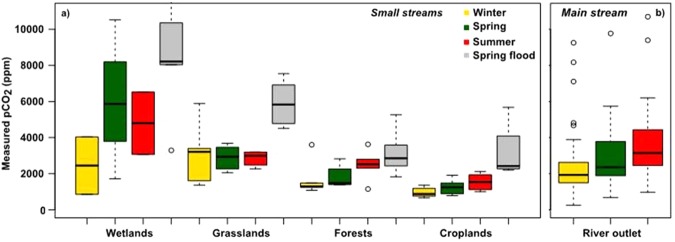
Boxplots of pCO_2_ assembled as function of the land uses and seasons investigated. The lower, intermediate and upper parts of the boxes represent respectively the 25^th^, 50^th^ and 75^th^ percentiles and the empty circles represent the outlier values. (**a**) pCO_2_ measured in stream waters (order 1 to 4) draining wetlands, grasslands, forests and croplands during the 2016 and 2017 field campaigns (hydro-climatic conditions are listed in Table 1). (**b**) pCO_2_ calculated from existing bi-monthly pH, total alkalinity and water temperature data at the outlet of the Seine River (Poses station) from 2013 to 2015 and aggregated by the four seasons of interest (see Materials and Methods, pCO_2_ calculations) (Data source: AESN).

All samples were supersaturated in CO_2_ with respect to the atmosphere, regardless of river characteristics (small stream or main stream), the associated dominant land use and the season (Fig. 3a). pCO_2_ increased significantly i.e., by an average of 49% and 62% from winter to summer/autumn, in streams draining forests and croplands (*p* < 0.05, Wilcoxon signed-rank test, Fig. 3a). Values in grasslands did not typically follow this pattern (*p* > 0.05, Wilcoxon signed-rank test, Fig. 3a), and pCO_2_ remained rather stable at 2,900 ppm, whereas pCO_2_ was the highest in wetlands (*p* < 0.05, Kruskal-Wallis test), especially in spring and summer/autumn (>4500 ppm).

For each of the four seasons monitored, pCO_2_ average decreases ranked as follows: wetlands > grasslands > forests > croplands (*p* < 0.05, Kruskal-Wallis tests, Fig. 3a). At the outlet of the Seine River, the main stream drains composite land uses where pCO_2_ averages were found to be equivalent to those measured in small streams draining grasslands (Fig. 3b).

In the lower Seine River, which is highly impacted by urbanization and associated treated WWTP effluents, a 69% increase in pCO_2_ was observed from winter (December to February), to spring (March to June) to summer/autumn (June to November) with limited dilution by the discharge (mean discharge in winter: 1030 m^3^ s^−1^; spring: 580 m^3^ s^−1^; summer/autumn: 270 m^3^ s^−1^) (Fig. 3b, Table 1).

However, during the late spring flood (1500 m^3^ s^−1^ at Poses, the Seine river outlet), pCO_2_ averages increased in all the land uses (3100, 3200, 5900, 8400 ppm for croplands, forests, grasslands and wetlands, respectively) (Fig. 3a).

In the groundwater, pCO_2_ averaged 27,000 ppm at a yearly scale, but reached up to 65,000 ppm (summer/autumn 2016), i.e., a factor of 5 to 10 compared to surface waters.

**Figure 4:**
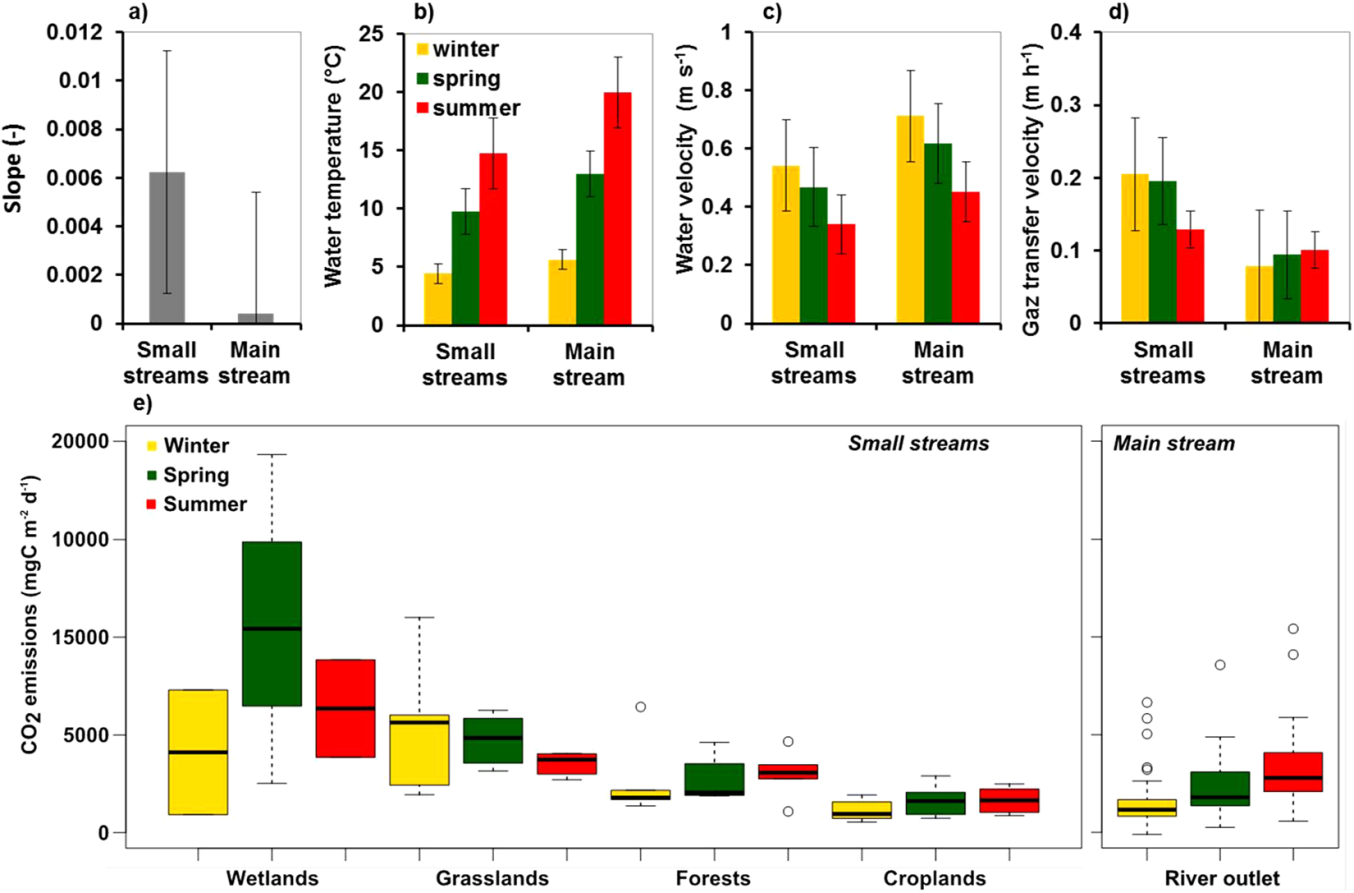
Comparison of the physical characteristics of small streams (orders 1 to 4) and of the main stream (orders 5 to 7) of the Seine River, averaged by season (excluding spring flood measurements): (**a**) slopes of the streams or rivers; (**b**) water temperatures; (**c**) water velocities; (**d**) gas transfer velocities; (**a**–**d**) Whiskers represent standard deviations; (**e**) boxplots of CO_2_ emissions assembled according to land uses. The lower, intermediate and upper parts of the boxes represent respectively the 25^th^, 50^th^ and 75^th^ percentiles and circles represent the outlier values.

According to the k_600_ equation selected (see Materials and Methods, equations 1–3), gas transfer velocity *(k*-values) was estimated from the slopes. *k-value* was higher for small streams (0.006 m m^−1^) than larger rivers (0.0004 m m^−1^) (Fig. 4a). Seasonal variations in water temperature increased from winter to summer/autumn (Fig. 4b), and velocities decreased from winter to summer/autumn (Fig. 4c). The resulting *k*-values ranged from 0.08 to 0.24 m h^−1^ with a decrease from small streams (*k* annual average = 0.19 m h^−1^) to larger rivers (*k* annual average = 0.09 m h^−1^) (Fig. 4d).

Using equation (2), the higher slopes found in small streams led to higher *k*-values (Fig. 4a and d). Additionally, in the small streams in the Seine River basin, the water velocity effect prevails over the seasonal *k* dynamics, while control by water temperature is greater in higher stream orders (Fig. 4b–d). During the spring flood event, the increase in the water discharge (and velocity) led to a greater increase in *k* in small streams than in larger rivers, respectively +26% (spring flood: 0.24 m h^−1^) and +11% (spring flood: 0.10 m h^−1^) compared to average spring *k*-values (small streams: 0.19 m h^−1^, larger rivers: 0.09 m h^−1^).

CO_2_ fluxes at the water-atmosphere interface (Fig. 4e) were estimated using the pCO_2_ measurements we made during our field campaigns (Fig. 3), CO_2_ saturation values that depend on water temperatures (see Table 1), atmospheric pCO_2_, and *k*-values estimations (Fig. 4d). The same seasonal pattern was observed for pCO_2_ and CO_2_ fluxes.

### Long-term pCO_2_ variability (1970–2015)

**Figure 5:**
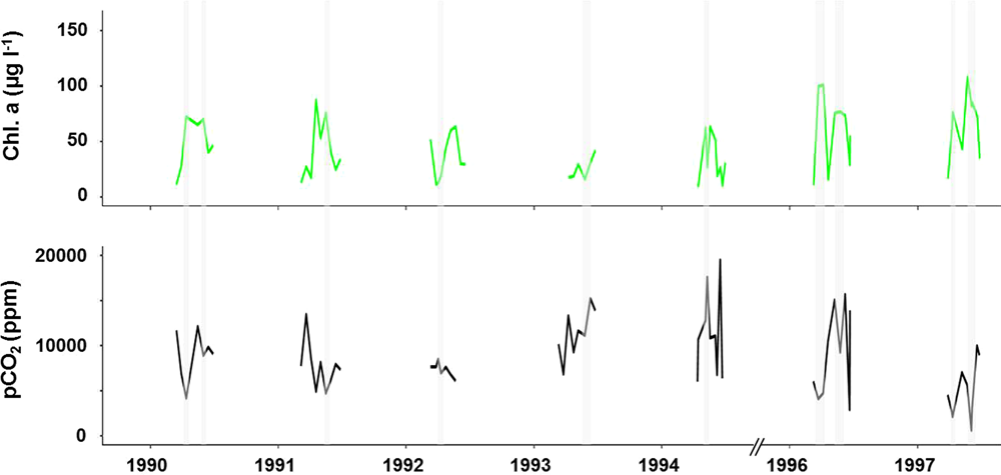
Calculated pCO_2_ dynamics during bloom events (Chl. a > 50 µg l^−1^) since the 1990s at the outlet of the Seine River, Poses (Data source: AESN).

Long-term analysis of French water authority (AESN) databases showed supersaturation of CO_2_ of the Seine River dating back to 1970 (98.5% data suggested supersaturation with respect to atmospheric equilibrium – pCO_2_ median = 3030 ppm; mean = 4765 ppm). From that period on, the Seine River has been a source of CO_2_ to the atmosphere even when frequent phytoplankton blooms occurred before wastewater treatment was improved. However, focusing on the bloom events (Chl. a >50 µg l^−1^, Fig. 5), we observed the opposite pattern between Chl. a and pCO_2_ dynamics, with depletion of pCO_2_ concomitantly with peaks of phytoplankton. This consumption of CO_2_ was not sufficient to cause undersaturation of CO_2_ in the river (Fig. 5).

**Figure 6:**
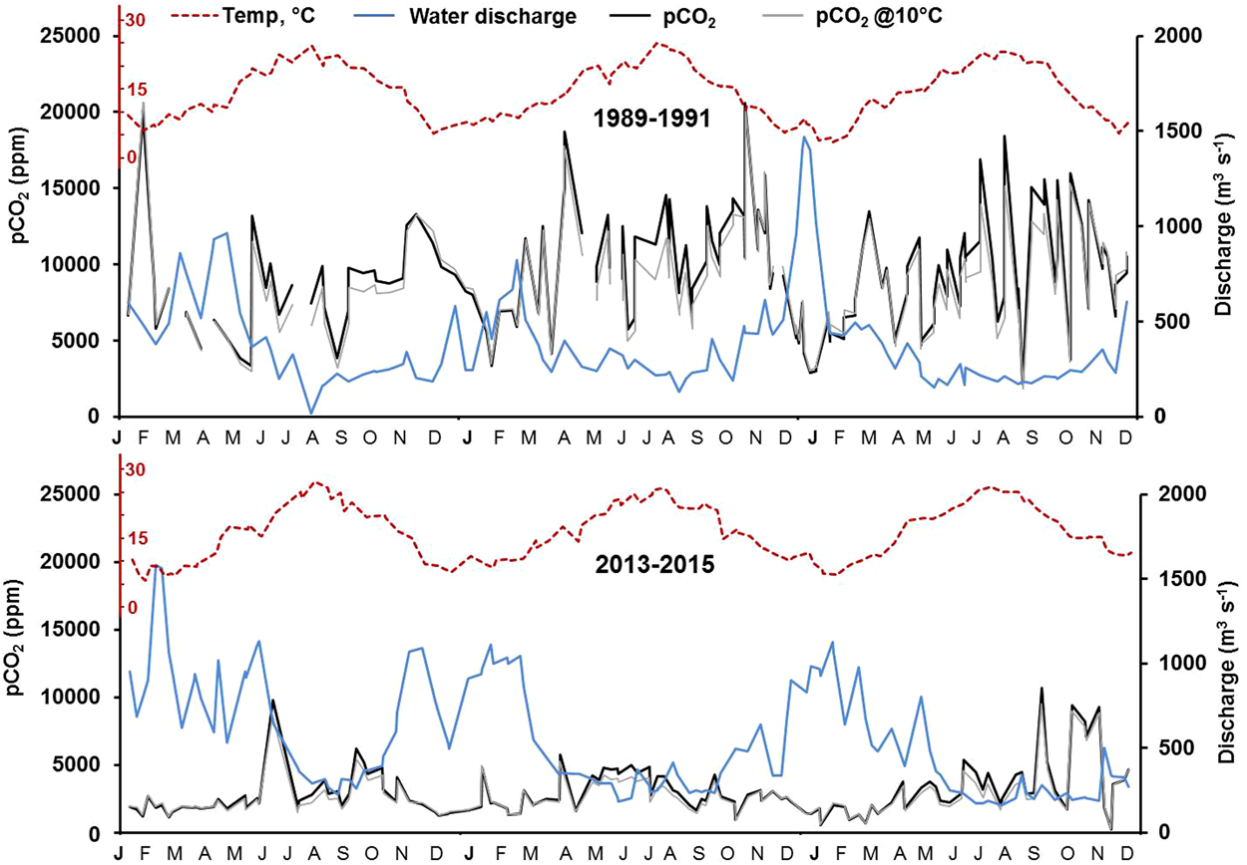
Seasonal variations in calculated pCO_2_ at the water temperaturer (pCO_2_) and at 10 °C (pCO_2_@10 °C) values compared with variations in water discharge and temperature values (at Poses) for the two periods: 1989–1991 (atmospheric pCO_2_ = 354 ppm) and 2013–2015 (atmospheric pCO_2_ = 399 ppm).

When we compared the two contrasted periods with respect to water sanitation and associated organic carbon releases, we found a similar range of temperature and discharge values and a seasonal pattern typical of temperate oceanic hydro-climatology regimes, i.e., high temperatures and low water in summer/autumn. The first of these two periods was however slightly drier than the second (on average 400 m^3^ s^−1^ vs. 545 m^3^ s^−1^, respectively) with no notable change in temperature (averaging 13.9 °C vs. 14.2 °C, respectively. We observed that pCO_2_ computed at both 10 °C and at water temperature were similar during winter but values at 10 °C were slightly lower than at water temperature during summer (Fig. 6). However, general trends of pCO_2_ did not change.

In contrast, pCO_2_ was reduced by a factor of 2.7 between the two periods (average 8250 ppm for the period 1989–1991 versus 3020 ppm for the period 2013–2015). These weak hydro-climatologic changes cannot explain the marked decrease in pCO_2_ at the river outlet between the two periods. We found no relationship between pCO_2_ and discharge at this time scale, despite a clear antiparallel trend for these two variables (Fig. 6).

**Figure 7:**
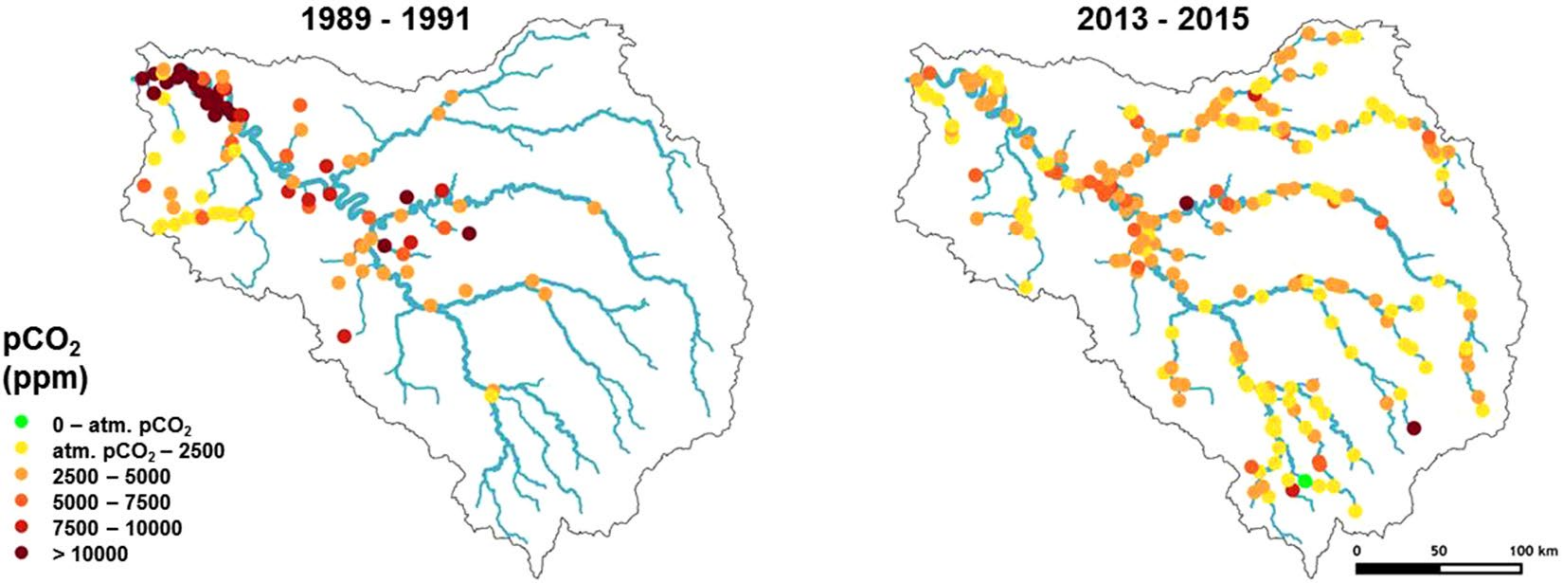
Spatial variations in calculated pCO_2_ averaged over the two periods 1989–1991 (with high organic pollution) and 2013–2015 (after wastewater treatment had been improved). Values are represented for Strahler orders superiors to 2 (Data source: AESN).

To further explore the recent decrease in pCO_2_, we assessed spatial variations in pCO_2_ at the scale of the whole Seine drainage network (Fig. 7). Although fewer measurements were available in the earlier period (1989–1991), the decrease in pCO_2_ between the two periods was obvious along the lower reach of the main stream of the Seine River, downstream of the Paris conurbation. In the recent period, the pCO_2_ of both the upstream parts of the drainage network and the main stream of the Seine River appear to be equally supersaturated (p > 0.05, Kruskal-Wallis tests, Fig. 7).

**Figure 8:**
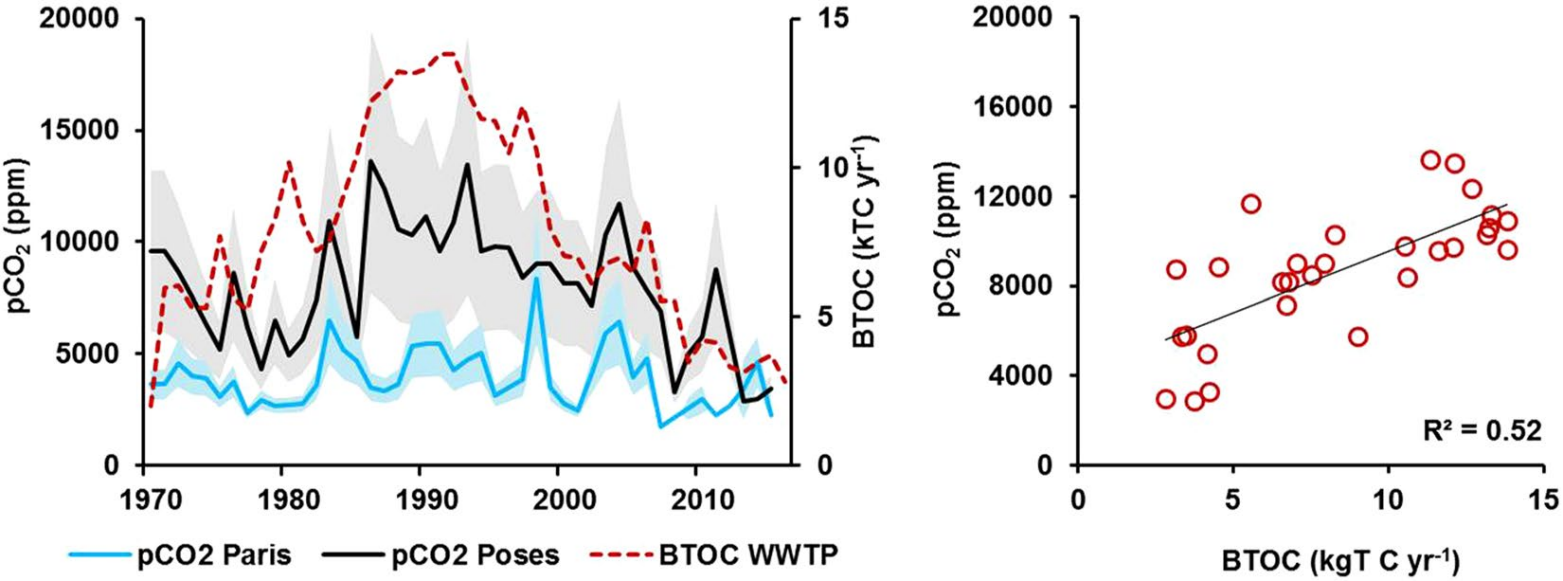
(**a**) Long-term variations in calculated pCO_2_ from 1970 to 2015 at two sites on the lower Seine River: at the entrance to Paris (blue curve) and at the river outlet at Poses downstream of the main WWTP SAV (black curve), the associated shaded areas represent the 95% confidence intervals (Data source: AESN). The red dashed curve represents the biodegradable total organic carbon fluxes (BTOC) discharged from the SIAAP WWTPs into the Seine River. BTOC was estimated from the relationship BTOC = 0.35 BDO (R² = 0.91, n = 23) established by Servais *et al*. (1999)^63^ which converts biological demand in oxygen (BDO, provided in Rocher and Azimi, 2017^64^) into BTOC; (**b**) Relationships between calculated pCO_2_ at Poses and BTOC from the SIAAP WWTPs.

Since the 1970s, upstream of the discharge of treated effluent from the SAV WWTP, the long-term trend of pCO_2_ values in Paris has varied around 5000 ppm (Fig. 8a). A few kilometers downstream, at the outlet of the Seine River at Poses (strongly influenced by Parisian wastewater discharges) pCO_2_ progressively increased to reach a maximum of 12,000 ppm in the 1990s, and then slowly decreased to present values of 3000–4000 ppm (Fig. 8a). This decrease in pCO_2_ was concomitant with changes in the fluxes of biodegradable total organic carbon (BTOC) discharged by the WWTPs of the Parisian conurbation managed and operated by the Greater Paris sanitation authority (French acronym SIAAP) after treatment (Fig. 8a). From the 1990s to 2015, the BTOC load decreased by 80% (from 13.8 to 2.8 kt BTOC yr^−1^) following treatment improvements on the SAV WWTP site, the construction of a new WWTP in 1991 and of three new WWTPs between 2005 and 2008, conjointly with improvement in treatment at existing plants. A positive linear relationship (R^2^ = 0.52, n = 29, p < 0.05) was found between annual pCO_2_ at the outlet of the Seine River (Poses) and BTOC fluxes from the SIAAP WWTPs (Fig. 8b).

### pCO_2_ environmental controls

**Figure 9:**
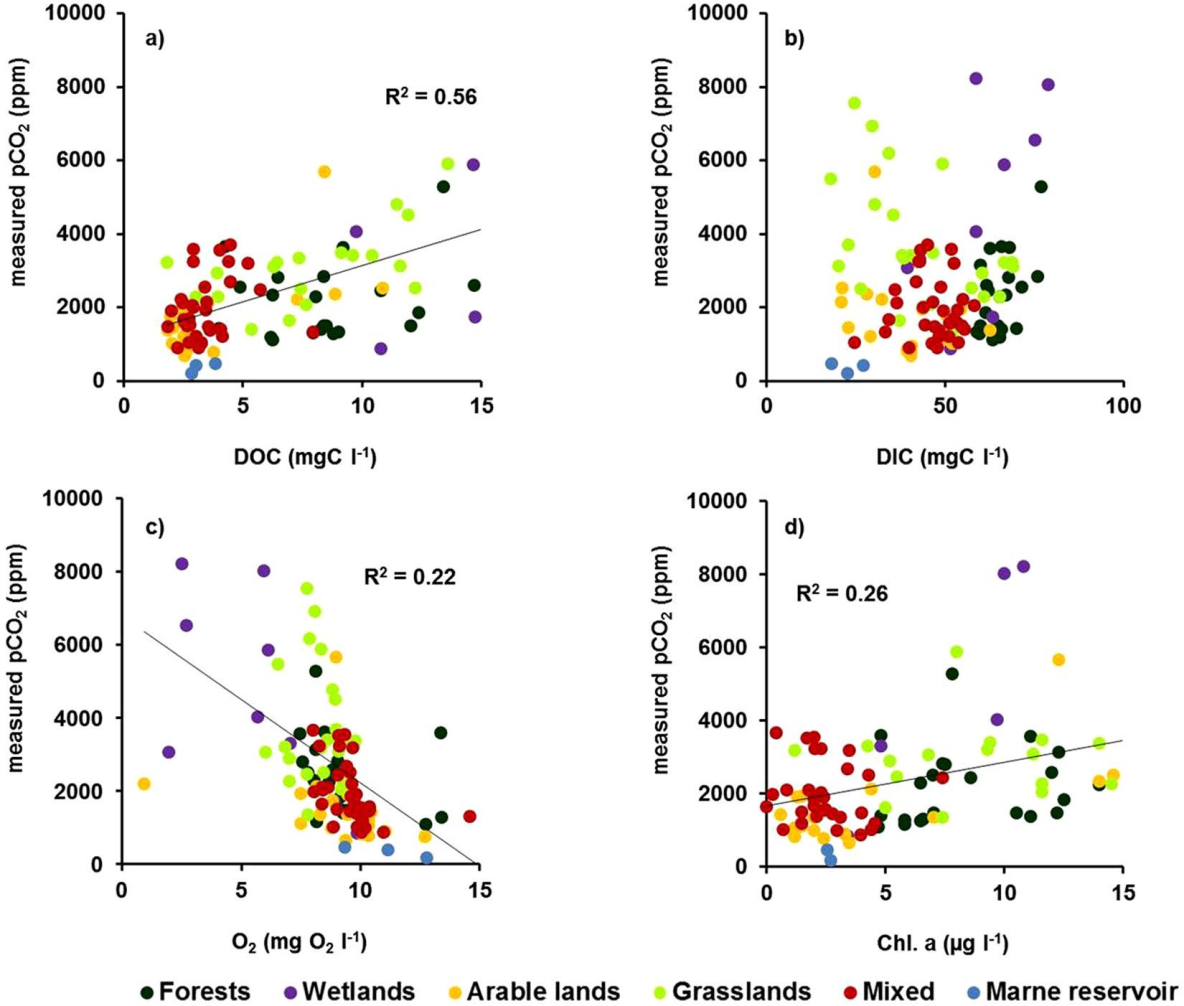
Relationships between measured pCO_2_ and surface water quality variables according to the different land uses sampled during the field campaigns: streams draining forests, wetlands, arable lands, grasslands, mixed in the main stream when no dominant land use could be identified, and the Marne reservoir. (**a**) pCO_2_ vs. dissolved organic carbon (DOC); (**b**) pCO_2_ vs. dissolved inorganic carbon (DIC); (**c**) pCO_2_ vs. dissolved oxygen (O_2_); and (**d**) pCO_2_ vs. chlorophyll a (Chl. a).

We found a positive linear relationship between pCO_2_ and DOC (R^2^ = 0.56, n = 119) (Fig. 9a). DOC measured in grasslands (DOC average: 10.3 mg L^−1^; SD: 5.8 mg L^−1^) and wetlands (DOC average: 21.0 mg L^−1^; SD: 14.6 mg L^−1^) showed wider and higher ranges of concentration compared to arable lands (DOC average: 3.8 mg L^−1^; SD: 2.7 mg L^−1^) (Fig. 9a). Generally, the ranges of DOC and pCO_2_ were lower in winter and higher in summer/autumn and during the spring flood. No relationship was found between pCO_2_ and DIC or nutrients (Fig. 9b, see Supplementary Material 2). The highest pCO_2_ and the lowest oxygen concentrations were measured in anoxic wetlands, whereas the opposite was found in the Marne River reservoir, and overall, a negative relationship between pCO_2_ and dissolved oxygen was observed for all land uses (R^2^ = 0.22, n = 120, p < 0.05, Fig. 9c). We also found a positive relationship between pCO_2_ concentrations and Chl. a concentrations (R^2^ = 0.26, n = 113, p < 0.05, Fig. 9d).

Interestingly, pCO_2_ values measured during the field campaigns in the Marne reservoir showed CO_2_ undersaturation with respect to the atmospheric equilibrium, averaging 360 ppm in the reservoir and 413 ppm in the air; slight supersaturation (457 ppm (reservoir) and 402 ppm (air)) was only observed in the late summer/autumn samples. Chl. a concentrations in the reservoir were low (mean: 2.6 µg l^−1^) and DOC level was around 3.2 mgC l^−1^, i.e., with no sign of eutrophication (Fig. 9a,d).

## Discussion

### pCO_2_ supersaturation of the Seine hydrosystem

Since the 1970s, the whole drainage network of the Seine basin has been supersaturated in pCO_2_ with respect to the atmospheric equilibrium. Supersaturation was observed for 98.5% of computed pCO_2_ as well as for the direct field measurements. These results are in agreement with those obtained in the lower reaches of other temperate rivers. In comparison to the mean of 3000 ppm at the outlet of the Seine basin, average pCO_2_ in the Meuse River (Belgium) on the period 2011–2014 was found equaling 2004 ± 912 ppm and all samples were also supersaturated in CO_2_ (min. 971 ppm)^14^. Such variations can be found within the Scheldt River estuary (Belgium) and measurements in five of its tributaries (Dender: 8300 ppm, Zenne: 5700 ppm, Dijle: 7252 ppm and Nete River: 6700 ppm, and an average of 9500 ppm for the lower Scheldt)^36^. Other Rivers as the Leyre (France) showed the same range of values (average: 4429 ppm, min.: 901 ppm max.: 23,047 ppm)^12^. At the global scale, pCO_2_ in streams and rivers have been averaged at 1600 ppm in a range of 132 to 11,770 ppm^2^. The wide range of pCO_2_ values in rivers were already mentioned with variations from 10 to 100 times the saturation value^37^.

Conversely, we measured undersaturation in the Marne reservoir with pCO_2_ below or near atmospheric equilibrium, in agreement with the results reported by Crawford *et al*.^38^ for river basins containing dam reservoirs. Riverine reservoirs have a higher residence time than the river itself, leading to particle sedimentation and a decrease in turbidity, conditions that favor primary production, i.e., consumption of CO_2_ and production of oxygen. During our field campaigns, we did not observe eutrophication conditions (Fig. 9d) or relationships between pCO_2_ and nutrients (see Supplementary Material 2). Without eutrophication of the reservoir, the biomass produced does not form an organic load that would – paradoxically – consume O_2_ and release CO_2_^39^.

pCO_2_ is known to be affected by metabolic processes related to nutrient availability^40^. In the Seine River, we could have expected a relationship with ammonium (NH_4_^+^) as activated sludge treatment releases dissolved organic carbon and high ammonium load^21^. However, no direct relationship was found with NH_4_^+^ or other nutrients, which shows the complexity of the controls on pCO_2_ mentioned below.

Because the main stream of the Seine River was known for its phytoplankton blooms before domestic wastewater was efficiently treated^21,29^, we analyzed bloom events (Fig. 5) to try and identify possible short periods of undersaturation. Despite the fact we found evidence for the opposite pattern between phytoplankton (Chl. a) and pCO_2_ for phytoplankton blooms above 50 µg Chl. a l^−1^, the Seine River waters remained supersaturated.

This result supports the assumption that other environmental variables actively control pCO_2_ in the Seine River.

### Hydro-climatic controls on pCO_2_

As shown in Fig. 6, seasonal pCO_2_ concentrations (in the long term) varied in parallel with temperature (i.e., with the highest values in summer/autumn) and opposite to hydrology. Such dynamics are typical for the temperate oceanic regime of the Seine River, with high discharge in winter and low discharge in summer/autumn^41^.

Hydro-climatic effects resulted from a combination of water temperature and hydrology leading to a seasonal increase in pCO_2_ and CO_2_ evasion fluxes (*fCO*_2_) from winter to summer/autumn^6,14^. Indeed, the hypothesis of control by water temperature is strengthened by the results of the field campaigns for different land uses with increasing pCO_2_ according to the season (pCO_2_ in winter < spring < summer/autumn), which can be interpreted as an enhancement of DOC mineralization whatever the land use. However calculating pCO_2_ at 10 °C, revealed that temperature effect on solubility is rather low. In addition, long term seasonal variations in pCO_2_ suggest possible control by hydrological regimes (high pCO_2_ in low flow periods). In fact, both water temperature and hydrological regimes (water velocity) contributed to the variations in the gas transfer velocity (*k-*values*)*, and the associated *fCO*_2_. Moreover, for both *k*-values and *fCO*_*2*_ we demonstrated opposite seasonal patterns in the upstream and downstream parts of the Seine River system, differences that could be more attributed to water velocities in small streams and water temperatures in higher stream orders (see equation 4, Fig. 4b–d). Higher k*-values* upstream, caused by higher turbulence, logically led to important CO_2_ outgassing compared to the lower k-values of the lower Seine River, down to its outlet.

The highest pCO_2_ values were measured during the exceptional flood when groundwater overflows may have reinforced pCO_2_ in the surface water. These high in-stream pCO_2_ levels were found concomitantly with high *k*-values (10–55% higher than levels measured in small streams in the other seasons) and would be expected to enhance CO_2_ evasion from rivers to the atmosphere. Similar effects of hydro-climatic conditions have also been observed in the tropics, e.g., in a large Amazonian river with a 20% higher outgassing of CO_2_ during extreme flood years than in other years^42^, and in the Zambezi River, with pCO_2_ up to twofold higher during the wet season^43^. Polsenaere and Abril (2012)^44^ compared two French streams and one river and observed that the stream with the highest concentration of CO_2_ also had the highest CO_2_ degassing flux.

Several authors have already suggested that climate change may alter the frequency and amplitude of flood events in the Seine River basin, with more extreme hydrological conditions^45–47^, so that pCO_2_ and CO_2_ evasion could increase in the future.

### Control of pCO_2_ by the soil organic carbon stock

Analyzing in-stream pCO_2_ measured in the various upstream land uses as a function of DOC underlined the importance of soil organic carbon stocks as a controlling factor. pCO_2_ and DOC were higher in streams draining wetlands and grasslands compared to those draining forests and croplands (Figs 3 and 9a). According to Arrouays *et al*.^48^, the organic carbon stocks in croplands are less than 4.5 kg C m^−2^, but reach nearly 7.0 kgC m^−2^ in grasslands and forests and around 9.0 kgC m^−2^ in wetlands. These values are consistent with the higher carbon sequestration rate of grasslands and wetlands:104 ± 73 gC m^2^ year^−1^ on average in Europe^49^. Thus, differences in pCO_2_ according to land use here can be explained by the drainage of different organic soils and subsequent POC and DOC mineralization depending on water circulation and temperature. This result is clearly illustrated by the flood event flushes during the growing season when DOC (spring flood DOC median: 11.44 mgC l^−1^) and pCO_2_ (spring flood median: 3297 ppm) reached their highest values.

In addition to the carbon leached from riparian zones and sediments, organic carbon can be leached from soils where spring biological activity had already built up a large quantity of biomass that is potentially subject to mineralization.

Organic carbon quality has also been shown to influence pCO_2_ in streams in the North Central European plains in Germany and Poland^50^, and Belgium^14,51^. The biodegradable fraction of DOC is usually around 25% in upstream waters but may decrease to 5% in winter, and may be 50% in treated effluents^16^ (Garnier *et al*., unpublished data). This supports lower observed pCO_2_ in winter and higher values linked to WWTP effluents. Increasing biological mineralization of land-based organic matter (OM) in response to a rise in temperature^52^ during the growing season, or increasing biodegradable DOC exports during high water or flood events^53^ appear to be two major driving factors of pCO_2_.

DOC and pCO_2_ inputs originating from land runoff and/or aquifer base flow (i.e., diffuse sources), are added to inputs from wastewater effluents (i.e., point sources, as wastewaters treated in specific plants are well localized).

### Control of pCO_2_ by urban effluents: long term evidence

Whereas hydro-climatic conditions and diffuse pCO_2_ and DOC inputs appeared to determine the seasonal variations in pCO_2_, long term changes in pCO_2_ observed over 1970–2015 suggested control by point sources, which are known to dominate observed changes in the Seine River^20,21,29,54^. Indeed, the long-term annual pCO_2_ values in the urbanized main stream of the Seine River strictly mirror variations in releases of urban OM by the largest WWTP of the Paris conurbation. Until 1990, the wastewater collection rate was intensified but wastewater treatment was not improved^55,56^. Later on, the OM from discharged effluent decreased, with a stepwise increase in the number of WWTPs within the Parisian conurbation^55^ and improved treatment processes, in response to both the Urban Wastewater Directive (1991/271/EC) and the Water Framework Directive (WFD, 2000/60/EC). In 2012, the technique changed from activated sludge to fully operational tertiary treatment (nitrification in 2007 followed by 70% denitrification in 2012)^21^, and improved water quality in terms of organic pollution and nutrients. Subsequently, that helped in reducing pCO_2_ concentrations and enabled to recover acceptable levels of dissolved oxygen^54^ downstream of major urban releases in the lower Seine an estuary. Because CO_2_ evasion pattern is likely to follow the pattern of pCO_2_ (see Figs 3 and 4), our results would support those reported by Prasad *et al*.^57^. Indeed, they compared the urbanized Anacostia waters to the lower Potomac waters flowing into the Chesapeake Bay (U.S.A.), and showed similar effect of organic matter and nutrients from urbanized landscapes on CO_2_ evasion.

Whatever the period studied during the last 45 years, point source organic pollution appeared to be the main determinant of pCO_2_ downstream of the treated effluents discharged into the lower Seine River. However, hydro-climatic conditions also influence pCO_2_. For example, with no significant seasonal variations in OM fluxes discharged as point sources, higher pCO_2_ concentrations in summer are explained by a low OM dilution rate during low waters and high temperatures.

### Limits of the approach

The measurements we took in 2016 and 2017 showed neutral or basic carbonate buffered waters and DOC average seasonal concentrations of 3.5 to 11.4 mgC l^−1^, excluding overestimation of calculated pCO_2_ linked to the contribution of organic acids to TA^12^. Abril *et al*.^12^ also emphasized the importance of accurate pH measurements. We think that the variability we found when establishing the relationship between measured pCO_2_ and computed pCO_2_ (Fig. 2) could be linked to the accuracy of pH measurements. As a result, the long-term pCO_2_ analyses were subject to similar variability (see the 95% confidence intervals in Fig. 8a). Nevertheless, the amplitude of pCO_2_ variations over the 45 years period enabled a robust analysis.

The choice of computing *k* using one of the equations provided by Raymond *et al*.^30^ could lead to bias. Indeed the equation was proposed based on measurements made on small streams (median depth, 0.28 m) and during low flow (median discharge, 0.54 m^3^ s^−1^). We took into account slope, water velocity -*discharge divided by the wetted cross section***-** and water temperature, but not other physical or environmental factors causing turbulence in streams, e.g., water turbidity, bed frictions, the direction and the intensity of wind, and chemical or bio-films^30,44^. Although there is need for direct measurements of *k* in higher stream orders to reduce uncertainties in flux calculations, *k*-values calculated for the Seine River range between 0.08 m h^−1^ (in winter for the main stem) and 0.21 m h^−1^ (in winter for streams). *k*-values and patterns found for the Seine River are in agreement with *k*-values estimated for other large rivers (e.g., in New England, on the Upper Mississippi and the Upper Colorado Rivers^30^). Raymond *et al*.^2^ who averaged the *k* of the entire drainage network (mixing large rivers and streams) by coastal segmentation and related catchment regions (COSCAT)^58^ provided an annual *k*-value of 0.22 m h^−1^ for the region including the Seine River (COSCAT 401), close to the ones we used for streams. As small streams (SOs 1–4) represent 91% of the surface area of the Seine drainage network (French water authorities, AESN), our *k*-values seem reasonable. In addition, main stem *k*-values calculated for the Seine basin are in the range of global estimations found by Raymond *et al*.^2^, (median: 0.22 m h^−1^, min.: 0.07 m h^−1^, max: 1.43 m h^−1^).

At this stage, it is not possible to quantify the apportionment of pCO_2_ originating from carbonated groundwater from that resulting from carbon mineralization or WWTP inputs. The modelling approach in progress should provide quantitative insights and δ^13^C-DOC/POC analysis could also be useful to identify the different sources of pCO_2_^44^.

## Conclusions

This study showed that since 1970, both small-order streams and urbanized downstream rivers in the Seine River basin have been supersaturated in CO_2_ and a source of CO_2_ to the atmosphere. CO_2_ supersaturation with respect to the atmospheric equilibrium appeared to be controlled differently in space (depending on land uses or on the location of the main WWTP effluent discharge) and over time (seasonal or interannual). CO_2_ supersaturation depended on complex interactions between land based and groundwater discharges (upstream diffuse sources), and urban pressures (downstream point sources) modulated by hydro-climatic factors.

In the small streams of the drainage network, in sparsely populated zones, the highest pCO_2_ in summer was shown to originate from mineralization (increasing with water temperatures) of organic carbon from diffuse sources including in-stream bottom sediments, riparian and/or terrestrial soils varying according to land uses. Hydro-climatic variations, especially water velocity in small streams greatly affected gas transfer velocity, and helped determine in-stream pCO_2_ (and evasion). During the exceptional flood event, high water discharges following a period of growth probably increased the DOC flushed from soils, leading to higher pCO_2_, especially in streams draining wetlands and grasslands. High pCO_2_ in streams may be also linked to high pCO_2_ of groundwaters that feed the surface water during low flow, and to the overflow of aquifers during floods, with particularly high pCO_2_.

Based on the 1970–2015 time series, point source organic pollution appeared to be the main driver of pCO_2_ in the lower Seine River, downstream of the main outlet of WWTP effluents, and whatever the period studied. pCO_2_ was highest in summer during low waters and high temperatures, and lower in winter when the discharged effluents were diluted. Despite the notable decrease in organic pollution following improvements in WWTPs since the 1990s, pCO_2_ has remained higher than atmospheric values, strongly suggesting the influence of carbonated groundwater.

In the next step, a CO_2_ budget of the Seine drainage network will help (i) quantify the role played by temperate human-impacted rivers in the global carbon budget, and (ii) estimate the amount of pCO_2_ point sources vs. diffuse sources. The present study also points to the need for high frequency and more spatially resolved pCO_2_ values and direct measurements of *k*. In addition, to anticipate the impact of climate change with the expected extreme hydrological conditions, further research is needed to understand the interactions between the terrestrial (soils and their land-use), and aquatic (hydrosystems^59^, groundwater discharges) compartments of watersheds.

## Electronic supplementary material

Supplementary Information

## Data Availability

The datasets generated during the current study are available from the corresponding author on reasonable request.
