# Supplementary material for: Seasonal and spatial variability of the partial pressure of carbon dioxide in the human-impacted Seine River in France
Source: Sci Rep. 2018 Sep 18;8:13961. doi: 10.1038/s41598-018-32332-2 (PMC6143529; doi:10.1038/s41598-018-32332-2)
Supplement: Supplementary file 1 — Supplementary Information [file 41598_2018_32332_MOESM1_ESM.pdf]

Supplementary information of the article:

Seasonal and spatial variability of the partial pressure of carbon dioxide in the human-impacted Seine River in France

A. Marescaux, V. Thieu, A.V. Borges, J. Garnier

Sampling stations	Latitude (WGS 84)	Longitude (WGS 84)	Land uses	Winter	Spring flood	Summer/ Automn	Spring
Melarchez	N 48°52'05.5''	E 003°11'38.4''	Agriculture	22/02/16	30/05/16	08/09/16	14/03/17
Theil	N 48°48'58.2''	E 003°06'43.3''	Agriculture	22/02/16	30/05/16	08/09/16	14/03/17
Avenelles	N 48°49'44.5''	E 003°07'03.4''	Agriculture	22/02/16	30/05/16	08/09/16	14/03/17
Tresmes	N 48°48'45.5''	E 002°59'31.3''	Agriculture	22/02/16	30/05/16	08/09/16	14/03/17
Ru Bourgogne	N 48°50'13.26	E 003°06'35.84	Agriculture	08/03/16	31/05/2016	14/09/16	15/03/17
Avenelles	N 48°50'23.40	E 003°08'22.27	Agriculture	08/03/16	30/05/2016	14/09/16	15/03/17
Forests 6	N 49°20'55.8"	E 002°54'08.6"	Forests	25/02/16	25/05/16	06/09/16	21/03/17
Forests 5	N 49°21'37.8"	E 002°52'52.6"	Forests	25/02/16	25/05/16	06/09/16	21/03/17
Forests 3	N 49°22'26.3"	E 002°48'67.9"	Forests	25/02/16	25/05/16	06/09/16	21/03/17
Forests 2	N 49°22'33.4"	E 002°48'40.9"	Forests	25/02/16	25/05/16	06/09/16	21/03/17
Forests 4	N 49°21'82.7"	E 002°48'32.5"	Forests	25/02/16	25/05/16	06/09/16	21/03/17
Forests 1	N 49°22'26.7"	E 002°47'41.0"	Forests	25/02/16	25/05/16	06/09/16	21/03/17
Grasslands 4	N 49°29'27.5"	E 001°38'96.1"	Grasslands	24/02/16	23/05/16	07/09/16	23/03/17
Grasslands 2	N 49°32'10.6"	E 001°34'54.4"	Grasslands	24/02/16	23/05/16	07/09/16	23/03/17
Grasslands 3	N 49°32'11.0"	E 001°34'55.0"	Grasslands	24/02/16	23/05/16	07/09/16	23/03/17
Grasslands 6	N 49°30'31.3"	E 001°40'14.0"	Grasslands	24/02/16	23/05/16	07/09/16	23/03/17
Grasslands 5	N 49°30'56.7"	E 001°39'18.1"	Grasslands	24/02/16	23/05/16	07/09/16	23/03/17
Grasslands 1	N 49°32'10.4"	E 001°34'40.3"	Grasslands	24/02/16	23/05/16	07/09/16	23/03/17
Der lake	N 48°36'15.1"	E 004°43'04.8"	Lake	NA	24/05/16	12/09/16	16/03/17
St Maurice	N 48°48'58.16"	E 002°25'27.35"	Mixed	22/02/16	30/05/16	12/09/16	14/03/17
Choisy	N 48°46'20.52	E 002°24'38.58"	Mixed	22/02/16	30/05/16	08/09/16	14/03/17
Bougival	N 48°52'53.85"	E 002°06'53.99"	Mixed	23/02/16	26/05/16	13/09/16	22/03/17
Conflans S.	N 48°59'22.49"	E 002°04'59.13"	Mixed	23/02/16	26/05/16	13/09/16	22/03/17
Porcheville	N 48°58'26.12"	E 001°48'19.15"	Mixed	23/02/16	26/05/16	13/09/16	22/03/17
Conflans O.	N 48°59'53.14"	E 002°04'17.64"	Mixed	23/02/16	26/05/16	13/09/16	22/03/17
Matougues	N 48°59'49.1"	E 004°14'29.8"	Mixed	NA	24/05/16	12/09/16	16/03/17
Dormans	N 49°04'31.3"	E 003°38'10.3"	Mixed	NA	24/05/16	12/09/16	16/03/17
St Maurice	N 48°48'58.16"	E 002°25'27.35"	Mixed	NA	24/05/16	12/09/16	16/03/17
Arrigny	N 48°37'19.6"	E 004°42'03.5"	Mixed	NA	24/05/16	12/09/16	16/03/17
Wetlands 1	N 49°22'27.7"	E 002°47'430"	Wetlands	10/03/16	25/05/16	06/09/16	21/03/17
Wetlands 2	N 49°22'21.0"	E 002°47'550"	Wetlands	10/03/16	25/05/16	06/09/16	21/03/17
Wetlands 5	N 49°21'44.6"	E 002°49'742"	Wetlands	10/03/16	25/05/16	06/09/16	21/03/17
Wetlands 3	N 49°22'22.7"	E 002°48'565"	Wetlands	10/03/16	25/05/16	06/09/16	21/03/17
Wetlands 4	N 49°21'12.9"	E 002°48'742"	Wetlands	10/03/16	25/05/16	06/09/16	21/03/17
G.Piezo 1	N 48°50'25.60"	E 003°06'14.76"	Groundwater	08/03/2016	31/05/2016	14/09/2016	15/03/2017
G.Piezo 2	N 48°50'20.60"	E 003°06'22.75"	Groundwater	08/03/2016	31/05/2016	14/09/2016	15/03/2017
G.Piezo 4	N 48°50'12.12"	E 003°06'32.58"	Groundwater	08/03/2016	31/05/2016	14/09/2016	15/03/2017
Ave. PZ1	N 48°50'23.48"	E 003°08'22.23"	Groundwater	08/03/2016	30/05/2016	14/09/2016	15/03/2017
Ave. PZ2	N 48°50'08.01"	E 003°08'43.03"	Groundwater	08/03/2016	30/05/2016	14/09/2016	15/03/2017
Ave. PZ3	N 48°49'47.38"	E 003°09'25.15"	Groundwater	08/03/2016	30/05/2016	14/09/2016	15/03/2017

Supplementary information 1. Location of the stations sampled during the field campaigns in 2016-2017. Measurements undertaken: carbon dioxide partial pressure, dissolved organic carbon, particular organic carbon, dissolved organic carbon, particular inorganic carbon, dissolved oxygen, total suspended solids, water temperature, pH, total alkalinity, silica, phosphate (PO_4^-), total phosphorus (Ptot), ammonium (NH_4^+), nitrite (NO_2^-), and nitrate (NO_3^-).

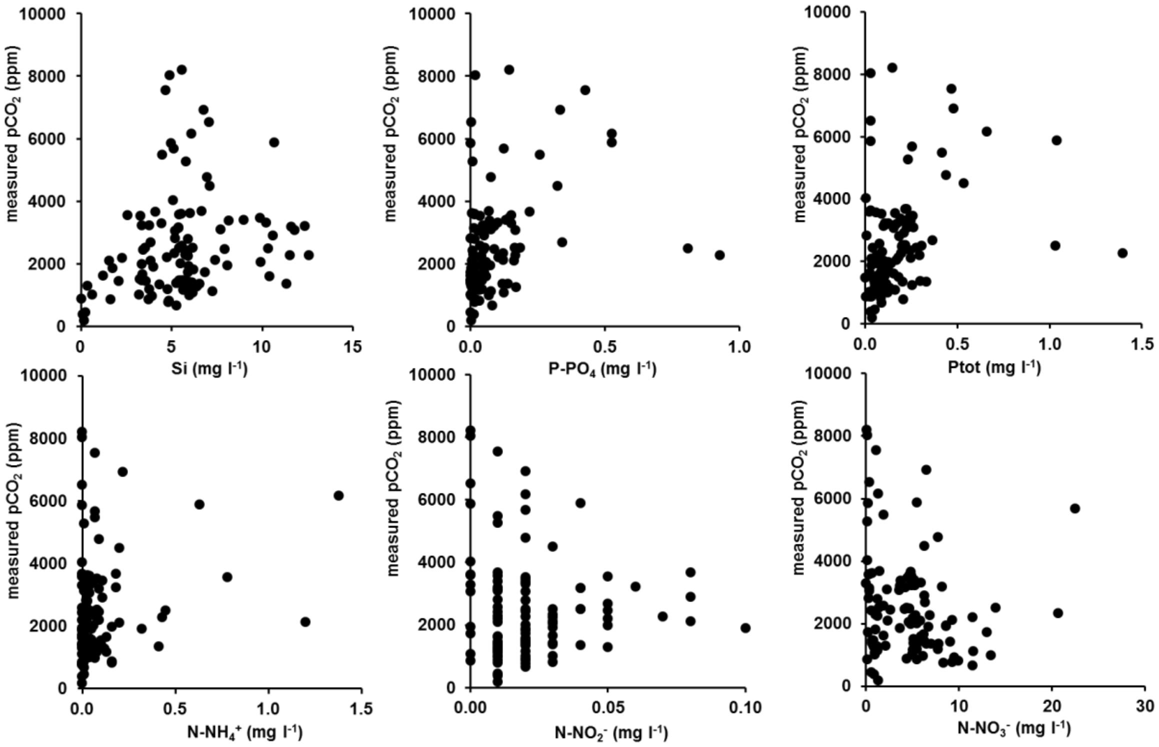

Supplementary information 2. Direct pCO_2 measured during the four field campaigns vs nutrients: silica (Si), phosphate (PO_4^-), total phosphorus (Ptot), ammonium (NH_4^+), nitrite (NO_2^-), and nitrate (NO_3^-).
